# Programmability of Co-antidot lattices of optimized geometry

**DOI:** 10.1038/srep41157

**Published:** 2017-02-01

**Authors:** Tobias Schneider, Manuel Langer, Julia Alekhina, Ewa Kowalska, Antje Oelschlägel, Anna Semisalova, Andreas Neudert, Kilian Lenz, Kay Potzger, Mikhail P. Kostylev, Jürgen Fassbender, Adekunle O. Adeyeye, Jürgen Lindner, Rantej Bali

**Affiliations:** 1 Helmholtz-Zentrum Dresden–Rossendorf, Institute of Ion Beam Physics and Materials Research, 01328 Dresden, Germany; 2 Technische Universität Chemnitz, Institute of Physics, 09107 Chemnitz, Germany; 3 Technische Universität Dresden, Department of Physics, 01069 Dresden, Germany; 4 Lomonosov Moscow State University, Faculty of Physics, 119991 Moscow, Russia; 5 University of Western Australia, School of Physics, 6009 Crawley, Australia; 6 National University of Singapore, Department of Electrical and Computer Engineering, 117576 Singapore

## Abstract

Programmability of stable magnetization configurations in a magnetic device is a highly desirable feature for a variety of applications, such as in magneto-transport and spin-wave logic. Periodic systems such as antidot lattices may exhibit programmability; however, to achieve multiple stable magnetization configurations the lattice geometry must be optimized. We consider the magnetization states in Co-antidot lattices of ≈50 nm thickness and ≈150 nm inter-antidot distance. Micromagnetic simulations were applied to investigate the magnetization states around individual antidots during the reversal process. The reversal processes predicted by micromagnetics were confirmed by experimental observations. Magnetization reversal in these antidots occurs *via* field driven transition between 3 elementary magnetization states – termed *G, C* and *Q*. These magnetization states can be described by vectors, and the reversal process proceeds *via* step-wise linear operations on these vector states. Rules governing the co-existence of the three magnetization states were empirically observed. It is shown that in an *n* × *n* antidot lattice, a variety of field switchable combinations of *G, C* and *Q* can occur, indicating programmability of the antidot lattices.

Magnetic antidot lattices are intriguing objects composed of a periodic array of holes (antidots) within an otherwise continuous magnetic film^1^,^2^,^3^,^4^,^5^. The periodicity of the antidot lattice translates to a periodic magnetization distribution in its ground state, which is disturbed by the application of external magnetic fields. Antidot lattices are therefore ideal testbeds to study the collective behaviour of non-trivial periodic distributions under the influence of external disturbances.

The periodicity of antidot lattices makes them viable for applications in magnonic devices, whereby the propagation of spin-waves can be influenced by the lattice geometry^6^. Spin-wave functionality can be dramatically enhanced in devices with programmable magnetization states^7^. Generally in such experiments the collective behaviour of the antidots has been considered – however the local variation of the magnetization around antidots, and their potential for programmability have rarely been taken into account^8^. Controllable switching between magnetization configurations is a desirable feature for devices; for instance in magnonics each configuration may correspond to a specific wave propagation mode thereby realizing a switchable spin-wave filter. Furthermore, antidot lattices possess continuous metallic paths making it possible to perform transport measurements. If the antidot lattice possesses several stable magnetization states, then each state may correspond to a specific magneto-resistance value^9^. To realize the occurrence of antidot lattices with stable magnetization configurations, and to achieve reliable field driven switching between these states; it is essential to understand magnetization reversal mechanisms in antidot lattices.

Several previous studies on magnetization reversal in antidots have focussed on the macroscopic aspects of the reversal mechanisms, for instance on the effects of lattice geometry and spacing on the coercivity and magneto-resistive properties^5^,^9^,^10^,^11^,^12^,^13^,^14^, as well as on the magnetization dynamics^15^,^16^,^17^,^18^. However, studies of the magnetization states of *individual* antidots within the lattice are rare.

The magnetization reversal behaviour of antidot lattices can be adjusted using a number of geometric parameters *viz.*, the antidot shape (square, elliptical or circular), the lattice geometry (square or hexagonal), the antidot diameter and inter-antidot spacing, and the thickness of the antidot lattice. For instance it has been reported that non-circular antidot geometries can contribute an additional anisotropic component to the magnetization reversal, and to exclude this factor we restrict ourselves to circular antidots^19^.

Lattice symmetry plays a major role in determining the reversal mechanism. Magnetization reversal in square lattices tends to proceed *via* the motion of domain walls along continuous magnetic channels of the [10] direction^20^. On the other hand, the reversal process in hexagonal and other lattice geometries is more complex^21^. The predictability of the reversal mechanism, at least for magnetic fields applied along the [10] direction and the avoidance of anisotropy contributions from the holes themselves lead us to confine this work to circular antidots within a square antidot lattice.

Within square antidot lattices, the influence of lateral geometry *i.e.*, the relation between antidot diameter, spacing and corresponding reversal behaviour has been reported in detail^22^. For a fixed antidot diameter, decreasing the inter-antidot spacing should leads to a transition from antidot like behaviour whereby the coercivity increases, to a transition regime and finally to a particle like behaviour where the magnetic material is separated and the coercivity decreases. It is desirable to achieve increased coercivity and thereby increase the stability of the observed magnetization configurations. According to literature, restricting to the antidot regime, while maintaining the thinnest possible distance between the antidot rims will maximize coercivity. Constraining the inter-dot spacing is expected to suppress the formation of domain walls. However for any potential application, it is necessary for the antidots to possess sufficient material to enable sensing of the magnetization states, either optically or by passing electrical currents.

Magnetization reversal mechanisms in square-lattice antidots have been studied *via* direct microscopic observations^23^,^24^,^25^. However, most studies on the reversal behaviour of magnetic antidot lattices consider lattice thicknesses of 10 to 25 nm. Increasing the lattice thickness further can lead to curling of the magnetization within the film depth, potentially approaching bulk like film behaviour and reducing the effect of the antidot pattern. Nevertheless, we increased the lattice thickness to 50 nm, which is a critical thickness for the occurrence of cross-tie domain walls in continuous Co films^26^,^27^. The formation of cross-tie domain walls leads to the appearance of vortex and anti-vortex like structures, possessing strong out-of-plane magnetization components at their centre. Magnetization reversal in antidot lattices of enhanced thickness have usually not been considered previously^28^.

We consider the magnetization states around individual antidots in Co-antidot lattices of a constricted inter-antidot distance, *w* ≈ 150 nm, and of thickness, ≈50 nm. Micromagnetic simulations were applied to investigate the magnetization states around individual antidots during the reversal process. The reversal processes predicted by micromagnetics were confirmed by experimental observations. Magnetization reversal in these antidots occurs *via* field driven transitions between 3 basic magnetization states – termed *G, C* and *Q*. These magnetization states can be described by vectors, and the reversal process proceeds *via* step-wise linear operations on these vector states. Rules governing the co-existence of the three magnetization states were empirically observed. It is shown that in an *n* × *n* antidot lattice, the *G, C* and *Q* states can occur in a variety of field switchable combinations, demonstrating programmability in the antidot lattices.

## Results

**Figure 1:**
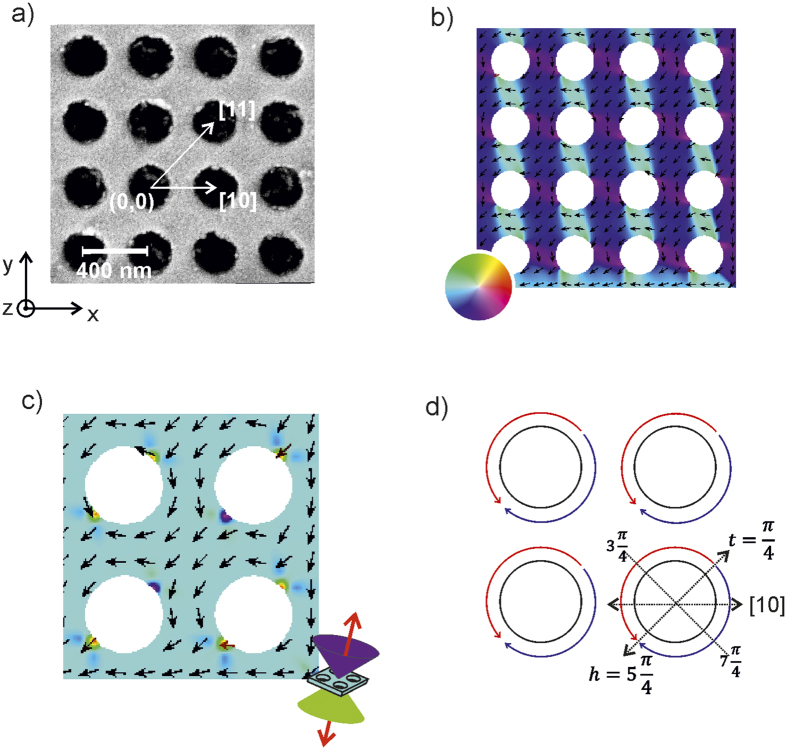
Antidot geometry. (**a)** SEM micrograph of the antidot lattice. Micromagnetic simulation of the antidot lattice, showing the color-coded (**b)** in-plane (**c)** close-up of the out-of-plane magnetization distributions. A schematic of the ground state is shown in (**d)**, indicating the clockwise and counter-clockwise directions along which the magnetization arcs around the antidot rims, with the two arcs with head-to-head, *h*, and tail-to-tail, *t*, intercepts respectively located at angular positions of 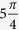 and 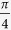. The coloured double-cone of (**c**) follows the same scheme as the red-green-blue-red colour wheel of (**b**), except that it indicates out-of-plane angular position.

Square lattice antidot arrays of 50 nm thick Co films consisting of 260 nm diameter holes separated by 415 nm centre-to-centre distance were studied, giving a lateral confinement, or minimum inter-antidot distance, *i.e.* the shortest distance between two antidot rims along the [10] direction, *w* ≈ 150  nm (Fig. 1a). The lattice was patterned over a 4 × 4 mm^2^ area. A scanning electron microscopy image of the antidot array is shown in Fig. 1a. Description of the sample preparation procedure has been published elsewhere^29^. Coercivities of the antidot lattice varied between 300 and 380 Oe depending on the direction of field w.r.t. the lattice symmetry, showing significant magnetic anisotropy induced by the antidot pattern. A control sample of continuous Co film shows coercivities of 5 and 18 Oe along the hard and easy axes respectively, showing that the magneto-crystalline anisotropy of the Co film material is negligible and consistent with a polycrystalline film structure.

At 50 nm Co thicknesses, it is possible to stabilize out-of-plane magnetization components, in the form of cross-tie domain walls^26^,^27^. The distance between the antidots has been constricted to suppress the formation of domain walls, particularly within the inter-antidot region. In continuous Co thin films, the distance over which the magnetization changes its direction *i.e.*, the domain wall width, is ≈50  nm^30^, which is still less than the ≈150 nm inter-antidot spacing. However the formation of domain walls is strongly influenced by the stray fields associated with the antidot geometry. Using micromagnetics, domain walls were simulated within the inter-antidot region with *w* ≈ 150 nm and were observed to be expelled as the magnetization relaxed.

Magnetization configuration in the ground-state *i.e.*, in the absence of applied magnetic field was calculated using micromagnetic simulations, shown in Fig. 1b. The magnetization was initialized along the [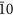] direction and then allowed to relax. The simulation was performed using the GPU based mumax^3^ code^31^ on an antidot array consisting of 10 × 10 unit cells without periodic boundary conditions. Figure 1b shows the in-plane magnetization orientation map of a section consisting of 4 × 4 unit cells, located at a corner of the 10 × 10 lattice. As seen in Fig. 1b,c, the magnetization tends to curl around the circumferences of the holes, forming magnetization-arcs. The arcs intercept at two points along the rim where the magnetization is forced out of the antidot plane. These intercepts will be used to describe the magnetization state around every antidot in the form of a vector, {*h, t*}, where *h* and *t* are the angular positions of the head-to-head and tail-to-tail intercepts respectively. Figure 1d depicts a ground-state magnetization where *h* lies at an angular position of 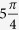 and *t* at 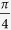. The magnetization state in Fig. 1d can be denoted in the form {*h, t*} as 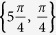, which we abbreviate to {5, 1} for convenience, making *h* = 5 and *t* = 1. Inspecting the magnetization distribution we observe that *h* and *t* always lie at positions that are odd multiples of 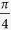 *i.e.*, the arcs always intercept at positions 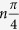 where *n* = 1, 3, 5 and 7. From symmetry, there exist four ground states that are energetically degenerate of the form *G*_*ht*_, where |*h* − *t*| = 4. We define switching between the four *G*_*ht*_ states as a magnetization reversal event.

**Figure 2:**
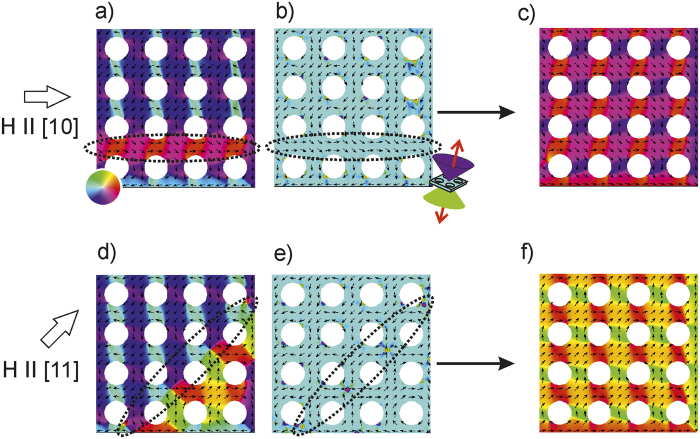
Micromagnetic simulations of magnetization reversal. Beginning with the ground-state distribution shown in Fig. 1b, reversal was simulated for magnetic field applied along the (**a)** [10] direction resulting in the switched ground-state in (**c)**. Similarly, reversal under a magnetic field applied along the [11] direction (**d)** switches the antidot lattices to the ground state shown in (**f)**. Dotted ellipses show the regions undergoing the reversal process. Distribution of the perpendicular magnetization component during reversal are shown in (**b,e)** for magnetic field applied respectively along the [10] and [11] directions. Simulations were performed on a 10 × 10 lattice and the results shown here are from the same 4 × 4 corner of the lattice. The applied magnetic fields are 40 mT in (**a,b**) and 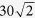 mT in (**d,e**). The coloured double-cone of (**b**) follows the same scheme as the red-green-blue-red colour wheel of (**a**), except that it indicates out-of-plane angular position.

Figure 2 shows a micromagnetic simulation of the magnetization reversal from an initial *G*_51_state. A magnetic field is applied parallel to the [10] and [11] directions respectively. With field applied along the [10] *i.e.*, H ll [10], reversal commences at the edges of the 10 × 10 lattice. Figure 2a shows a partially reversed situation stable under an applied field of 40 mT, where the moments within a continuous magnetic path bound by antidots on either side have rotated towards the applied magnetic field. The switched magnetic moments lie within the dotted ellipse shown in Fig. 2a,b. As the field is increased, the remaining continuous magnetic paths parallel to the [10] are also switched. Finally when the field is released the system relaxes to the nearest ground state, where the magnetization around the antidots can be described by a vector, *G*_73_ (Fig. 2c).

Similarly, when H is applied parallel to the [11] the reveral process commences at two of the corners of the 10 × 10 lattice. In a real system defects act as nucleation sites for magnetization reversal. In an ideal system at 0 K, domain nucleation commences at points of broken symmetry such as at the corners of the antidot lattice. Magnetization reversal commences close to the corners of the 10 × 10 lattice and progresses towards the centre, with the reversal front expanding at every field step. By focusing at the corners of the antidot lattice where the reversal process is initiated, we are able to observe the formation of the reversal front at the microscopic scale without interference from neighbouring domain walls. Figure 2d,e show a partially reversed situation at a lattice corner under an applied magnetic field of 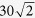 mT. The reversal front *i.e.*, the interface between the reversed and unreversed regions has been encircled by the dotted ellipse. The reversal front may also be viewed as a set of propagating domain walls periodically separated by antidots. With increasing H the reversal front moves diagonally across the sample, and as the field is released the lattice relaxes to a *G*_15_ ground state (Fig. 2f).

Note that for the intermediate reversal states shown above, for the antidots within the dotted ellipses, the magnetization around the antidots does not correspond to a ground state. For H ∥ [10], in Fig. 2a,b, it is seen that the magnetization arcs are not of equal lengths, rather of 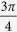 and 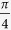 distribution around the antidots. Consequently, |*h* − *t*| is 2 instead of 4, and we generalize these states as *Q*_*ht*_. Similarly, for H ∥ [11] in Fig. 2d,e, the magnetization around the antidots at the reversal front is neither *G* nor *Q*. Instead the magnetization arcs complete a full circumference such that *h* = *t*. We define this magnetization state as a 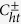, where the + sign defines a counter-clockwise magnetization circulation.

For each of the intermediate states above *i.e.*, for H ∥ [10] and H ∥ [11], the perpendicular component of the magnetization has been plotted in Fig. 2b,e, respectively. A distinctive feature of magnetization reversal with H ∥ [11] is the appearance of regions with perpendicular magnetization at the reversal front (Fig. 2e). These regions of perpendicular magnetization (anti-vortices) are positioned away from the antidot rims, and appear to be associated with 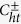 states only, as they are not observed with the field applied along the [10] forming rows of *Q* but no *C* states. Later it will be shown that magnetization reversal processes can be described by linear operations on *G, C*, and *Q* states.

The predicted differences in the reversal mechanisms for the magnetic field applied along the [10] and [11] directions were tested experimentally using Kerr Microscopy for mapping the in-plane magnetization component, as well as with Magnetic Force Microscopy (MFM) for observing the out-of-plane magnetization component during the reversal process. There is a significant difference in the resolution achieved by the above methods, with Kerr Microscopy at ~500 nm, whereas MFM at ~50 nm.

**Figure 3:**
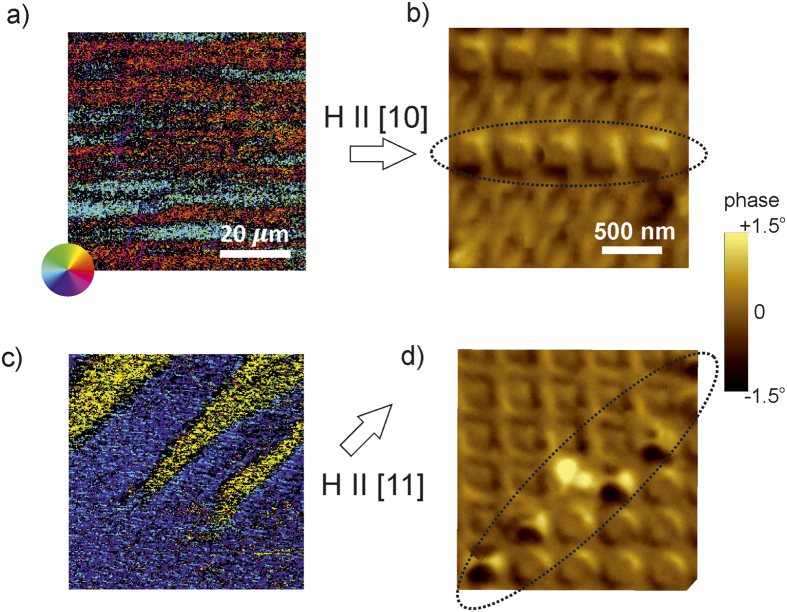
Imaging of the magnetization reversal process. (**a)** Kerr microscopy and (**b)** Magnetic Force Microscopy (MFM) images acquired while applying a magnetic field applied along the [10] direction and similarly, (**c)** Kerr- and (**d)** MFM- images acquired while applying the magnetic field along the [11] direction. Dotted ellipses show the regions undergoing the reversal process. The images were acquired in applied fields of (**a**) 33.5 (**b**) 45 (**c**) 30 and (**d**) 42 mT.

As seen in Fig. 3a applying the field along the [10] direction, causes the magnetization reversal to traverse along the [10], in agreement with the simulations. The MFM image of the process observed at a field of 40 mT shows rows of reversed regions along the [10], for instance the row indicated by the dotted ellipse in Fig. 3b. The colour scale shows the phase shift of the scanning tip tapped at 83 kHz, which is correlated with the out-of-plane stray fields emerging from the sample magnetization. The structural periodicity of the antidot lattice translates to a periodic stray field distribution observed here. The reversing regions seen in Fig. 3b show a higher contrast, suggesting a higher concentration of out-of-plane magnetization components occurring in these regions as compared to that of the ground state. Although the imaging resolution is not sufficient to conclusively prove the occurrence of the *Q* state in the revering regions, the enhanced phase shift helps distinguish the magnetization of these reversing regions from the *G* state.

Applying the field along the [11] direction (Fig. 3c) results in the formation of elongated domains aligned longitudinally with the applied field direction. The averaged magnetization orientation of the reversing domains is in agreement with the simulation shown in Fig. 2e, broadly confirming the expected differences in the reversal mode. A reversal front is observed at an applied field of 43 mT using MFM, shown in Fig. 3d. The reversal front runs diagonally from bottom-left to top-right and has been indicated by a dotted ellipse. The unreversed region lies above the diagonal and the reversing region is situated below. The reversing region shows enhanced phase shift suggesting the occurrence of non-*G* states, in line with the simulations. Significantly, located exactly at the reversal front are regions of strong contrast, which are consistent with the reversal front predicted by the micromagnetic simulations. The contrast in these regions is a composite of dark as well as light, indicating a complex magnetization structure whereby the stray field lines are emerging from the sample surface, as well as piercing it. The predicted magnetization configuration of these regions, as seen in Fig. 2e, shows that magnetic moments pointing out of the lattice surface are surrounded by magnetic moments of the opposite orientation. Although *C*-states could not be directly observed since they should possess weak out-of-plane stray fields, the periodic location of the out-of-plane contrast regions along the diagonal, coinciding with the reversal front, is a strong indication for the presence of such states.

## Discussion

**Figure 4:**
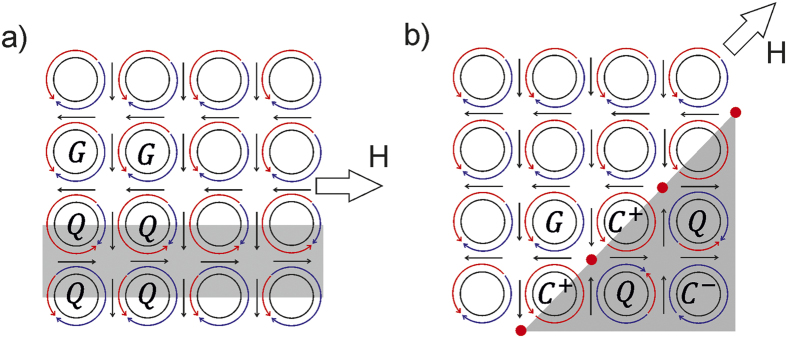
Schematic view of the magnetization reversal. The schematic shown in (**a)** corresponds to the reversal shown in Fig. 2a,b with the applied field parallel to the [10] direction and the schematic in (**b)** shows the reversal state in Fig. 2d,e with the applied field parallel to the [11] direction. Regions where the magnetization is reversing have been shaded grey. Red points mark regions possessing out-of-plane magnetization components. Arrows show the average inter-antidot magnetization orientation.

Having obtained empirical evidence of the occurrence of the various magnetization states, we now view the reversal in terms of *G, Q*, and *C* states. A schematic of the magnetization reversal in the [10] direction is shown in Fig. 4a. The application of H ∥ [10] leads to the transformation of rows of initially *G*- to *Q*-states. A *Q*_*ht*_ state can be obtained by linear vector addition on a *G*_*ht*_, and for the case shown in Fig. 4a:


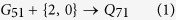


For the reversal process to be completed, the lattice relaxes to the *G*_73_. This is conveniently achieved by a second complementary linear addition step:


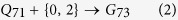


Equations (1) and (2) describe the repositioning of the *h* and *t* intercepts and consequent re-distribution of magnetization arcs around the antidot rims. Sequentially applying the above equations to antidot rows completes the reversal process of the antidot lattice.

The case of magnetization reversal along [11] can also be considered in a similar way. As mentioned previously, at the reversal front there exist antidots possessing a full circulation of the magnetization *i.e., C*; at the reversal front shown in Fig. 4b these states are 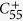. The reversal proceeds by vector addition:


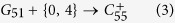


The reversed ground state is achieved by a further addition of {4, 0}:


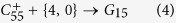


**Figure 5:**
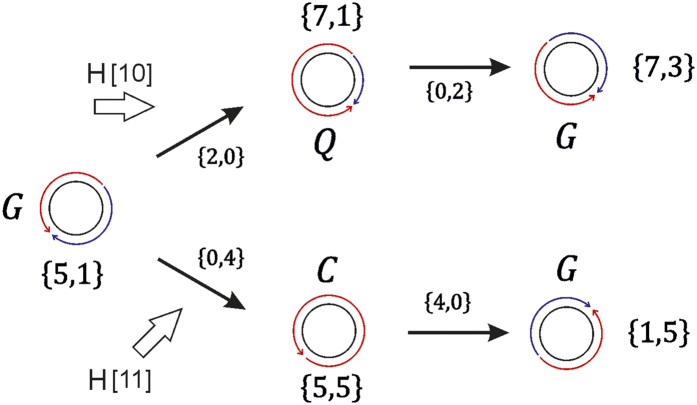
Magnetization reversal around a single antidot. Paths for magnetization reversal with field applied parallel to the [10] (upper path) and [11] (lower path) directions.

The two field direction dependent magnetization reversal paths are summarized in Fig. 5. The upper half of Fig. 5 shows the *G* → *Q* → *G* path taken when the applied magnetic field is parallel to the [10], and the lower half shows the *G* → *C* → *G* path that applies for field direction along the [11]. Whereas the *G* → *Q* → *G* reversal path with field along [10] describes the magnetization reversal process generally observed in antidot lattices^23^,^24^, observations of the *G* → *C* → *G* are rarer. The difference may lie in the thickness of the antidots studied here: antidot lattices reported in literature are usually of ~20 nm thicknesses. To test this, micromagnetic simulations were also performed for antidot lattices of exactly the same planar geometry but of 25 nm thickness, and the formation of the 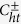-states was not observed. Instead the magnetization reversal follows a complex path consisting of the magnetization circulation breaking into several segments and the formation of vortices in the inter-antidot regions, which cannot be described by a simple mathematical model. Similarly one can expect complex reversal mechanisms in antidot lattices that are thicker than 50 nm whereby the magnetization can relax within the film depth, disabling the effects of lateral confinement. A study of square-shaped antidots patterned onto 50 nm thick Fe films also found resemblance of the magnetization configurations during reversal observed here^28^.

Implicit in the use of {*h, t*} to describe the magnetization states is that the *h* and *t* intercepts always occur as a single pair around an antidot rim. The exception is the 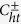, where there are no intercepts. Since at every *h* and *t* the magnetization tends to point out of the film plane, stray field minimization will impose a limit on the number of intercepts. Empirical rules governing the magnetization states of neighbouring antidots can be interpreted from the simulations on the given 10 × 10 antidot lattice system. Firstly, the magnetization within the antidot rim-to-rim separation does not contain domain walls. Although the rim-to-rim separation here is 150 nm compared with the typical domain walls of 50–70 nm widths observed in 50 nm thick Co films^32^,^33^, the curvature of the antidot rims can direct the magnetization towards a fixed direction, as observed here and in other reports on dense antidot lattices^10^. Secondly, within a given unit cell, the average magnetization of the inter-antidot regions (indicated by straight arrows in Fig. 4) follow a 2-in-2-out configuration. This is similar to the spin-ice rules observed in rectangular arrays of freestanding nanostructures, except that in the case of antidots the magnetic regions are connected making it difficult to realize rule violations or the so-called frustrated states^34^. The spin-ice rule is also observed at the lattice edges, where it is 2-in-1-out or 1-in-2-out.

It is worth noting that reversal in freestanding square rings of magnetic material follow paths that are similar to those described in Fig. 5 35. Furthermore, *C* and *Q* states were stabilized in the square rings even at zero applied field. This suggests that in the above antidot lattices, where we have shown that the *C* and *Q* states occur during the reversal process, it may be possible to stabilize these states in remanence as well. From the programmability point of view it is useful to investigate the coexistence of stabilized *G, C* and *Q* states in lattices with a limited number of antidots.

We tested for the existence of stabilized *C* and *Q* states using micromagnetic simulations on a 2 × 2 lattice of antidots. Such a four-unit system possesses 15 possible states composed of *G, C* and *Q*. For instance for H ll [11], stable *CQCQ, GCQC* can be identified at the reversal front (Fig. 4b). Similarly, blocks of *GGQQ* as well as *QQQQ* are observed during reversal along the [10] direction (see Fig. 4a).

The above magnetization configurations are seen in a 2 × 2 blocks that are part of a much larger lattice. This raises a question as to whether the stray fields from the edges of a free-standing 2 × 2 lattice will distort the configurations, or even cause deviations of the spin-ice like rules. This was tested by considering a free-standing 2 × 2 lattice and applying fields along various directions. Simulations reproduced several of the above configurations by application of the magnetic field. Typically, applying magnetic fields along the [11] results in combinations of *C* and *Q* states whereas fields along [10] leads to a mix of *Q* states. In some cases minor distortions were observed due to edge effects, which can likely be removed by further geometric refinements. It is clear that a free-standing *n* × *n* antidot lattice possess 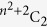 possible configurations, a large fraction of which may be realizable in experiments. The number of accessible combinations can be enhanced by applying local magnetic fields, for instance by current lines placed in the vicinity of the antidots – thereby programming the magnetization configurations by electrical pulses.

## Conclusions

In antidot lattices with constricted inter-antidot spacing to expel domain walls and of thicknesses that are close to critical for stabilized out-of-plane magnetization components, the magnetization reversal process reduces to transitions between three basic magnetization states - *G, C*, and *Q* that exist around individual antidots. An *n* × *n* antidot lattice of optimized geometry can be used as a highly re-programmable magnetic array. We envisage applications in magnonic devices where each configuration is translated into distinct spin-wave propagation modes. In electrically contacted antidot lattices, the magnetization distribution can also influence anisotropic magneto-resistance where electrical resistance can be tuned in discrete steps. Programmable antidot lattices may therefore possess dual magnonic and magneto-resistive functions, which is a valuable asset in device applications.

## Methods

### Antidots Preparation

Large area (4 mm × 4 mm) antidot lattices with circular holes arranged into a square matrix were defined on commercially available Si substrates using deep ultraviolet lithography at 248 nm exposing wavelength. Thereafter a 50 nm Co film was deposited using *e*-beam deposition at a rate of 0.2 Å/s in a chamber with a base pressure of 4 × 10^−8^ Torr. This was followed by an ultrasonic assisted lift-off process. The antidot array consists of 260 nm diameter holes separated by 415 nm centre-to-centre spacing between neighbouring holes. Details of the fabrication process are described in ref. 29.

### Magnetic Imaging

The imaging of the local magnetization direction was carried out by quantitative Magneto Optic Kerr effect based microscopy, here referred to as Kerr-microscopy, at 100× magnification. Due to the magneto optic Kerr effect, the polarization axis of the incident (linearly polarized) light is rotated (Kerr rotation) when being reflected at the surface of the magnetic sample. The rotation angle sensed by the Kerr-microscope is proportional to the in-plane magnetization component parallel to the plane of incidence leading to a pronounced contrast for magnetic domains magnetized parallel and antiparallel to the plane of incidence. To calibrate the Kerr image, the sample is saturated by an external magnetic field and the local contrast corresponding to the magnetic orientation is taken as the saturation intensity. In order to achieve the full 2D information about the local magnetic orientation, two Kerr images were taken with perpendicular sensitivity axes. A vector map is obtained such as shown in Fig. 3 with the HSV colors corresponding to the local angle of the magnetization.

Magnetic force microscope (MFM) equipped with low magnetic moment tips (PPP-LM-MFMR, Nanosensors) was used. In MFM the variation of magnetic force between the tip and surface is detected and the MFM image represents the distribution of the normal component of the stray field from the sample surface. The distance between tip and surface (lift height) was kept at 10 nm.

### Micromagnetic Simulations

Micromagnetic simulations are performed using the MuMax3 software package^26^. The 4 μm × 4 μm antidot lattice was discretized into 1024 × 1024 × 11 cells, resulting in a cell size of 4.05 nm × 4.05 nm × 4.5 nm. The saturation magnetization M_s_ = 1400 kA/m and the exchange constant A = 30 pJ/m were chosen according to literature values. As an example, for simulating the reversal along the [11] direction, the initial state was set to (−1, −1, 0), which corresponds to complete saturation along the [11] axis. The relaxed state of the system was obtained by integrating the Landau-Lifshitz-Gilbert equation with a large Gilbert damping constant α = 1. The hysteresis loop then was calculated applying the magnetic field along the [10] or [11] axis with a step size of 5 mT and minimizing the total energy of the magnetic system.

## Additional Information

**How to cite this article**: Schneider, T. *et al*. Programmability of Co-antidot lattices of optimized geometry. *Sci. Rep.* **7**, 41157; doi: 10.1038/srep41157 (2017).

**Publisher's note:** Springer Nature remains neutral with regard to jurisdictional claims in published maps and institutional affiliations.
